# On the Synergistic Catalytic Properties of Bimetallic Mesoporous Materials Containing Aluminum and Zirconium: The Prins Cyclisation of Citronellal

**DOI:** 10.1002/chem.201002909

**Published:** 2011-01-21

**Authors:** Selvedin Telalović, Anand Ramanathan, Jeck Fei Ng, Rajamanickam Maheswari, Cees Kwakernaak, Fouad Soulimani, Hans C Brouwer, Gaik Khuan Chuah, Bert M Weckhuysen, Ulf Hanefeld

**Affiliations:** [a] Gebouw voor Scheikunde, Technische Universiteit DelftJulianalaan 136, 2628 BL Delft (The Netherlands); [b] The Center for Environmentally Beneficial Catalysis, The University of Kansas1501 Wakarusa, Bldg. B, Lawrence, KS 66047 (USA); [c] Department of Chemistry, National University of SingaporeKent Ridge, Singapore 119260 (Republic Singapore); [d] Department of Chemistry, Anna UniversityChennai 600025 (India); [e] Department of Materials Science and Engineering, Maritime and Materials Engineering, Technische Universiteit DelftMekelweg 2, 2628 CD Delft (The Netherlands); [f] Inorganic Chemistry and Catalysis Group, Debye Institute for Nanomaterials Science, Utrecht University3584 CA Utrecht (The Netherlands)

**Keywords:** Brønsted acids, heterogeneous catalysis, Lewis acids, mesoporous materials, synergy

## Abstract

Bimetallic three-dimensional amorphous mesoporous materials, Al-Zr-TUD-1 materials, were synthesised by using a surfactant-free, one-pot procedure employing triethanolamine (TEA) as a complexing reagent. The amount of aluminium and zirconium was varied in order to study the effect of these metals on the Brønsted and Lewis acidity, as well as on the resulting catalytic activity of the material. The materials were characterised by various techniques, including elemental analysis, X-ray diffraction, high-resolution TEM, N_2_ physisorption, temperature-programmed desorption (TPD) of NH_3_, and ^27^Al MAS NMR, XPS and FT-IR spectroscopy using pyridine and CO as probe molecules. Al-Zr-TUD-1 materials are mesoporous with surface areas ranging from 700–900 m^2^ g^−1^, an average pore size of around 4 nm and a pore volume of around 0.70 cm^3^ g^−1^. The synthesised Al-Zr-TUD-1 materials were tested as catalyst materials in the Lewis acid catalysed Meerwein–Ponndorf–Verley reduction of 4-*tert*-butylcyclohexanone, the intermolecular Prins synthesis of nopol and in the intramolecular Prins cyclisation of citronellal. Although Al-Zr-TUD-1 catalysts possess a lower amount of acid sites than their monometallic counterparts, according to TPD of NH_3_, these materials outperformed those of the monometallic Al-TUD-1 as well as Zr-TUD-1 in the Prins cyclisation of citronellal. This proves the existence of synergistic properties of Al-Zr-TUD-1. Due to the intramolecular nature of the Prins cyclisation of citronellal, the hydrophilic surface of the catalyst as well as the presence of both Brønsted and Lewis acid sites synergy could be obtained with bimetallic Al-Zr-TUD-1. Besides spectroscopic investigation of the active sites of the catalyst material a thorough testing of the catalyst in different types of reactions is crucial in identifying its specific active sites.

## Introduction

Synergy between different types of catalytic sites is a much sought after objective. Synergy might occur between Lewis and Brønsted acid sites in monometallic materials or might be induced by the application of two different active metals. In this respect, the increased activity of hydrothermally treated faujasite-type Y zeolites in catalytic cracking reactions has intrigued scientists for decades. Already very early, the synergy between extra-framework aluminium, Lewis acid sites formed due to partial release of aluminium from the framework upon hydrothermal treatment and Brønsted acid sites was suggested.[1] Enhanced acidity of the catalyst was explained by direct coordination of Lewis acid sites to the Brønsted acid site. Very recently, a different type of mechanism for Brønsted–Lewis acid synergy was proposed based on NMR experiments and DFT calculations: Lewis acid sites in the form of extra-framework aluminium coordinate to the oxygen atom nearest to the Brønsted acid site, though there is no direct interaction between them.[2]

Besides the above-mentioned synergy between Brønsted and Lewis acid sites in zeolites, there are also other relevant examples found in mesoporous materials. Immobilisation of different lanthanide triflates inside the pores of siliceous SBA-15 led to different Brønsted/Lewis ratios and corresponding catalytic activity in Friedel–Crafts acylation of naphthalene with *p*-toluoyl chloride.[3] The synergy found for these catalytic systems was explained through the confinement of large molecules, such as lanthanide triflates inside the pores of SBA-15, causing significant physical perturbation of the surface hydroxyl groups of the host matrix giving rise to the formation of certain Brønsted type surface acid sites.

Most common and widespread examples of synergy are those found in different bimetallic catalysts used in several important reactions. Synthesis of bimetallic catalysts, such as amorphous SnO_2_–ZrO_2_, so as to achieve synergistic effects has led to improved catalysts for SO_2_ reduction produced in the direct sulfur recovery process (DSRP).[4] Steam or autothermal reforming of hydrocarbons is an important source of H_2_ for proton-exchange, membrane fuel cells (H_2_-PEMFC). However, reformed gas contains considerable amounts of CO leading to poisoning of the fuel cell. Pt and Pd mutually immobilised on perovskite oxide showed higher activity and stability in the water gas shift reaction in order to reduce CO compared to their monometallic counterparts.[5] The same effect was observed by applying CuO–CeO_2_ instead of individual oxides (CuO and CeO_2_) or their physical mixtures.[6] The properties of light-metal hydrides, which are interesting for their high hydrogen storage capacity, were improved by the addition of Fe and Zr through enhanced H_2_ desorption kinetics.[7] Fe-Ce-ZSM-5 catalysts showed high NO conversion in the selective catalytic reduction of NO with NH_3_ in a wide temperature window and even in presence of H_2_O and SO_2_ relative to Fe-ZSM-5 or Ce-ZSM-5.[8]

An alternative approach to studying the synergy between Brønsted and Lewis acid sites, as seen in the industrially important Y zeolites, is to synthesise a bimetallic mesoporous catalyst. This approach necessitates the partial replacement of aluminium inside an aluminosilicate material possessing both Brønsted and Lewis acid sites by a transition metal generating exclusively Lewis acid sites. Framework incorporated transition metals with different electronic properties from aluminium would affect the strength of the Brønsted acid sites already present. Three-dimensional mesoporous materials lend themselves particularly for such an approach as diffusion effects are virtually none existent and if synergy between acid sites does exist, it would improve the catalytic activity of three-dimensional mesoporous materials to that of zeolites. Thus diffusion limitations would be reduced without loss of acidity based activity, relative to zeolites. This bimetallic approach making use of mesoporous MCM-41 materials has been explored for Zn–Al and Ce–Al combinations and yielded indications for synergy; however, conclusive results have not yet been obtained and this necessitates the exploration of other suitable mesoporous matrices.[9, 10] However, very recently synthesised Fe–Al bimetallic catalysts using MCM-41 or SBA-15 as supports proved to possess a synergistic effect in oxidation of benzyl alcohol.[11, 12]

TUD-1 is such a porous solid, as it is a well-established mesoporous material with a three-dimensional structure, tuneable pore size distribution and surface area reaching values of 900 m^2^ g^−1^.[13, 14] The main advantage of TUD-1 concerning the study of Brønsted–Lewis acid synergy is that it is excellent for framework incorporation of different metals.[15–17] This high degree of framework incorporation is due to the complexing agent used in the synthesis of TUD-1, triethanolamine. It forms atrane complexes with different metals (M), thus ensuring incorporation as isolated metals rather than metal oxide clusters. By employing this approach, a wide range of M-TUD-1 with framework incorporated metals can be prepared and Al-TUD-1 materials possessing both Brønsted and Lewis acid sites have been applied in the Lewis acid catalysed Friedel–Crafts alkylation of phenol.[18] Zr-TUD-1 on the other hand possesses exclusively Lewis acid sites due to a high degree of metal incorporation inside the TUD-1 matrix.[17] By combining both metals inside the TUD-1 matrix it should become possible to fine-tune the ratio of Lewis and Brønsted acid sites due to the different types of acidity of the metals.[19] Thus Al-Zr-TUD-1 should be a showcase material with adjustable Brønsted and Lewis acidity, ideal to investigate whether synergy between these two types of acidity really exists and can be exploited for fine tuning catalytic activity.

This is the topic of this article: Al-Zr-TUD-1 has been used to investigate the presence of synergy between Brønsted and Lewis acid sites by performing a systematical variation of aluminium and zirconium inside the TUD-1 matrix with constant Si/M ratio instead of increasing the weight percentage of one metal, while keeping the weight percentage of the second constant. Bimetallic mesoporous catalysts were compared with their monometallic counterparts Al-TUD-1 and Zr-TUD-1 by using both spectroscopic methods and catalytic conversions. It will be shown that the synergistic effect between different Brønsted and Lewis acid sites in bimetallic mesoporous catalysts is very specific, substrate as well as reaction mechanism dependent.

## Results and Discussion

**Figure 1:**
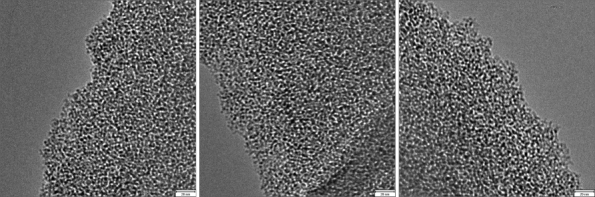
HR-TEM images of Al-Zr-TUD-1 materials with a Si/M ratio of approximately 25 varying in their Al:Zr ratio: Al-Zr-1.5:1 (left), Al-Zr-1:2 (middle) and Al-Zr-4.3:1 (right). The bars represent 20 nm.

The mesoporous nature of Al-Zr-TUD-1 samples is evidenced by the high-resolution transmission electron micrographs (HR-TEM) and representative images are given in Figure 1. All samples exibit a sponge-like structure typical for TUD-1. HR-TEM analysis proves the framework incorporation of Al and Zr in the TUD-1 matrix. No crystalline nanoparticles of ZrO_2_ or Al_2_O_3_ were detected, suggesting that both metals were incorporated into the framework as expected. This is in line with earlier results for monometallic Zr-TUD-1 and Al-TUD-1 with Si/M ratio of 25.[17, 18] Accordingly XRD analysis (Figure S1 in the Supporting information) further confirmed the mesoporous character of Al-Zr-TUD-1.

N_2_-physisorption measurements obtained at 77 K (Figure S2 and S3 in the Supporting information) and the results from elemental analysis (ICP-OES) are summarised in Table 1. The surface area of the three samples varies from 689 to 877 m^2^ g^−1^ and is not correlated with the amount of the different metals present. Pore size (*d*_p, BHJ_) and pore volume (*V*_P, BHJ_) are also not correlated to the type of metal present in the Al-Zr-TUD-1 samples. The Si/Zr ratios present in calcined samples differ from those in the initial synthesis gel. The more zirconium present in the sample, the higher is the deviation from the initial synthesis gel Si/(Al+Zr) ratio of 25, maximum obtained Si/(Al+Zr) ratio of 30 for the Al-Zr-1:2 sample. The same deviations were found during the synthesis of zirconium-containing aluminosilicate of BEA structure, Zr-Al-β. The Si/Al ratio of calcined Zr-Al-β was similar to the synthesis gel while the Si/Zr ratio was higher than the ratio of the synthesis gel.[20]

**Table 1:** Physicochemical and acidic properties of TUD-1-based catalysts

	*n*_Si_/*n*_M_[a]	*n*_Si_/*n*_Al_	*n*_Si_/*n*_Zr_	*n*_Si_/*n*_(Al+Zr)_	*S*_BET_ [m^2^ g^−1^]	*d*_P,BJH_ [nm]	*V*_P,BJH_ [cm^3^ g^−1^]	Total acidity [mmol NH_3_ g^−1^]	B/L ratio[b]
Al-TUD-1	25	26.6	–	26.6	956	3.7	0.95	0.40	2.41
Al-Zr-4.3:1	25	31	135	25	705	4.2	0.70	0.33	2.15
Al-Zr-1.5:1	25	47	69	28	686	4.6	0.85	0.32	2.05
Al-Zr-1:2	25	92	45	30	877	3.3	0.70	0.38	3.21
Zr-TUD-1	25	–	25	25	764	8.8	1.23	0.69	–

[a] Synthesis mixture ratio of silica to the total amount of metal incorporated.

[b] Determined by dividing the area under the Lewis acid region (1460–1440 cm^−1^) and the Brønsted acid region (1557–15 378 cm^−1^) found in spectra recorded at 200°C.

**Figure 2:**
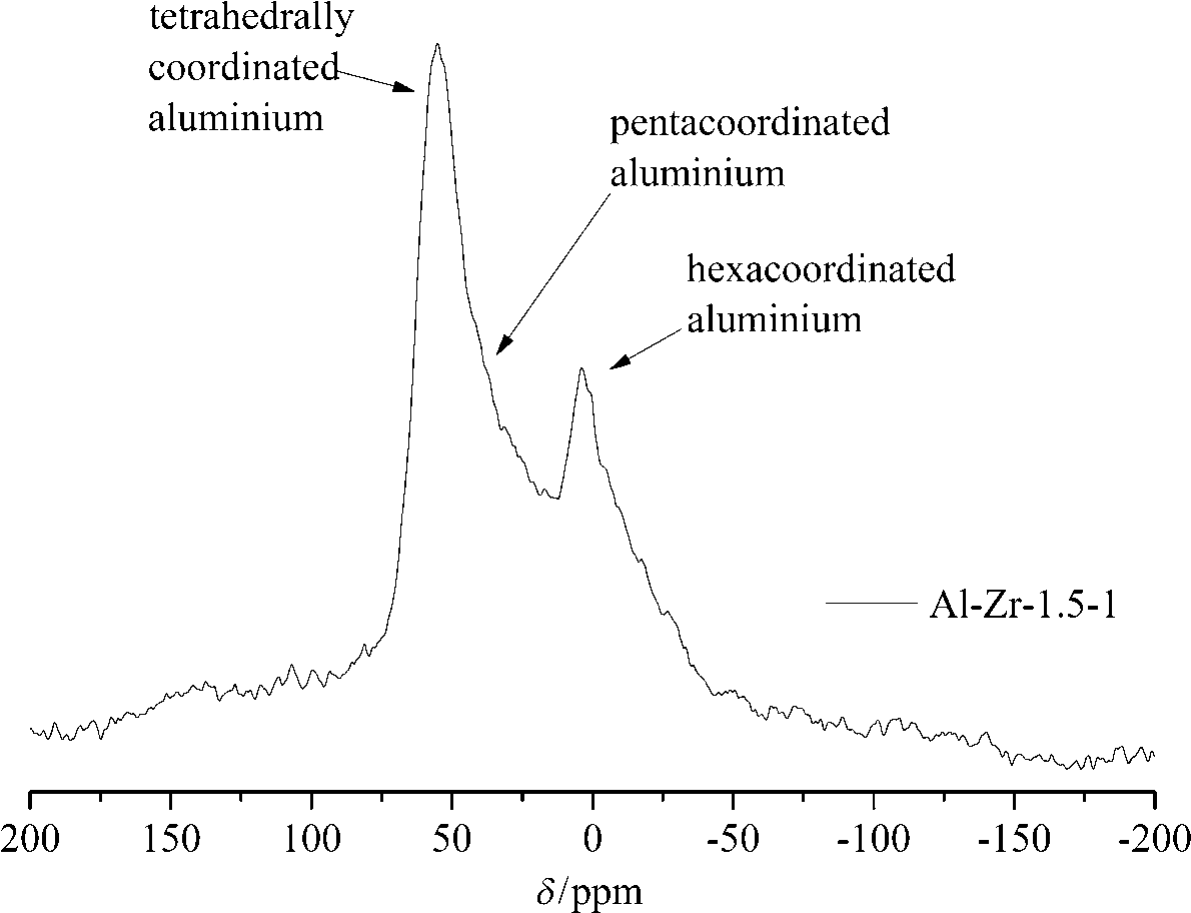
^27^Al MAS NMR spectrum of Al-Zr-1.5:1 catalyst, representative for all Al-Zr-TUD-1 materials with Si/M ratio of approximately 25.

The ^27^Al-NMR spectra of the three bimetallic catalysts are similar and reveal that around 50 % of aluminium is tetrahedrally incorporated, around 30 % is hexa-coordinated aluminium and is assigned to extra-framework aluminium responsible for Lewis acidity and finally around 20 % of aluminium is penta-coordinated (Figure 2). The Al in Al-Zr-TUD-1 induces therefore both Brønsted and Lewis acid sites.

From the XPS measurements, the binding energies for Si 2p and O 1s photoelectron lines resemble those found in silicon dioxide (SiO_2_). The spectra of O 1s, however, can be deconvoluted in five species for mono- and bimetallic TUD-1 catalysts (Table 2). The National Institute of Standards and Technology (NIST) X-ray photoelectron spectroscopy database give a value of the O 1s binding energy of 532.9±0.9 eV for SiO_2_ and 530.6±0.7 eV for both Al_2_O_3_ and ZrO_2_.[21]

**Table 2:** The XPS binding energy [in eV] of the O 1s, Al 2p, Si 2p and Zr 3d photoelectrons and position of the Auger lines for Al, Si and Zr

	Si-TUD-1	Zr-TUD-1	Al-TUD-1	Al-Zr 1:2	Al-Zr 1.5:1	Al-Zr 4.3:1
Al 2p	–	74.1	75.1	74.9	74.4	74.8
Si 2p	103.5	103.5	103.5	103.5	103.5	103.5
Zr 3d_5/2_	–	183.7	184.1	183.7	183.6	183.9
Zr 3d_3/2_	–	186.2	186.6	186.1	186.0	186.30
O 1s (1)	–	528.45	528.45	528.56	528.57	528.33
O 1s (2)	–	530.24	530.34	530.39	530.34	530.16
O 1s (3)	531.92	531.92	531.92	531.92	531.92	531.92
O 1s (4)	533.01	533.01	533.01	533.01	533.01	533.01
O 1s (5)	534.18	534.18	534.18	534.18	534.18	534.18

The Si-TUD-1 containing Si–O bonds only, has three O 1s peaks with a main peak always present at 533.0 eV. This main peak is flanked by two peaks with binding energies of 531.9 and 534.2 eV respectively (Table 2, Figure S4 in the Supporting Information) The binding energy of O 1s around 534 eV is due to the presence of air borne species such as water vapour, carbon dioxide or remaining template. An alternative explanation of the three peaks suggests that in Si-TUD-1, an amorphous mesoporous material, three prominent configurations of Si atoms bonded to an O atom are present.

Monometallic as well as bimetallic TUD-1 catalysts have O 1s binding energies that have a lower value than that of silicon oxide (Table 2). Here, the electronegativity of the Al (*χ*_Pauling_=1.61) and Zr (*χ*_Pauling_=1.33), as opposed to Si (*χ*_Pauling_=1.90), renders the oxygen atom to be more ionic.[22] The mutual repulsions of the electrons in the oxygen ion reduces the O 1s_1/2_ binding energy.

The presence of incorporated metal ions in the mesoporous framework apparently induces two spectral features at about 528.5 and 530.3 eV respectively. The latter peak can be ascribed to the “normal” ionic compounds Al_2_O_3_ or ZrO_2_.[21] It is also very clear from the spectra that it does not make a difference whether Al or Zr is involved (Figure S4 in the Supporting Information). They produce nearly the same spectra. The nature of the Al and Zr species is only derived from the binding energies of the metallic species. The presence of the ionic species does not affect the fraction of the flanking peaks too much (i.e., O 1s (3) and O 1s (5) in Table S1 in the Supporting Information), but reduces the central peak remarkably (i.e. O 1s (4)). This indicated that Al and Zr are incorporated in the TUD-1 framework at the expense of the Si–O bonds.

The binding energy of about 183.7±0.1 eV of Zr 3d_5/2_ is higher than the values reported for ZrO_2_[21] and corresponds to the values found in complex zirconium oxides as reported for ZrSiO_4_ and zirconium silicate alloy thin films.[23, 24] All zirconium found is incorporated into the SiO_2_ framework.

**Figure 3:**
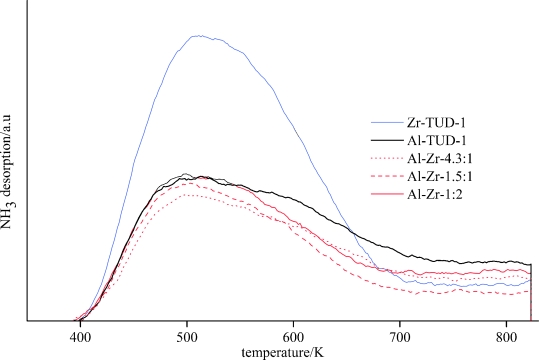
NH_3_-TPD profile of different TUD-1 catalysts with Si/M ratio of approximately 25: Zr-TUD-1, Al-TUD-1, Al-Zr-4.3:1, Al-Zr-1.5:1 and Al-Zr-1:2.

Based on NH_3_ temperature-programmed desorption (TPD) analysis (Figure 3) of different Al-Zr-TUD-1 catalysts, the acidity is of comparable order in all samples. The monometallic Zr-TUD-1 possesses a larger amount of weak acid sites and Al-TUD-1 has a larger amount of strong acid sites compared to the other catalysts (Table 1). Mutual incorporation of Al and Zr into TUD-1 leads to both a decrease of weak as well as strong acid sites and thus to a decrease of overall acidity.

**Figure 4:**
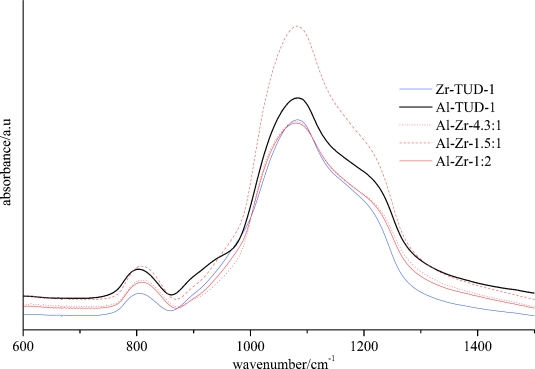
FT-IR skeletal spectra of different TUD-1 catalysts with Si/M ratio of approximately 25: Zr-TUD-1, Al-TUD-1, Al-Zr-4.3:1, Al-Zr-1.5:1 and Al-Zr-1:2.

IR spectra of a KBr pressed disc of three samples in the skeletal region (Figure 4) show typical bands at 1093 cm^−1^ and a shoulder at 1220 cm^−1^ due to asymmetric stretching vibrations of Si-O-Si bridges. The band at 798 cm^−1^ is caused by symmetric stretching vibration of Si-O-Si.[25] The signal for Si-OH or Si-O-M at approximately 970 cm^−1^ is not resolved.[23, 26, 27]

**Figure 5:**
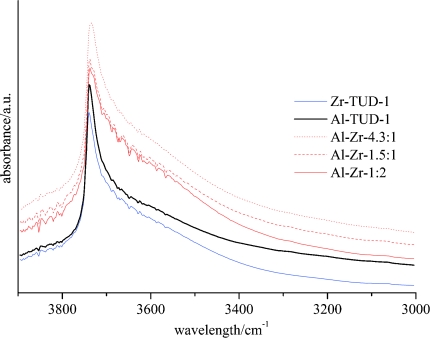
FT-IR spectra in OH region acquired at 400°C of different TUD-1 catalysts with Si/M ratio of approx 25: Zr-TUD-1, Al-TUD-1, Al-Zr-4.3:1, Al-Zr-1.5:1 and Al-Zr-1:2.

FT-IR of the OH region of the three bimetallic catalysts and those of Zr-TUD-1 and Al-TUD-1 (Figure 5) show a band centred around 3745 cm^−1^, asymmetric towards lower frequencies, commonly assigned to terminal silanol groups. However, there is a distinct difference between monometallic and bimetallic catalysts. Major bands centred at 3745 cm^−1^ for the bimetallic catalysts have higher values for the full width at half maximum compared to monometallic catalysts. This is an indication of higher degree of heterogeneity of different silanol groups inside Al-Zr-TUD-1 catalysts.

In addition to isolated silanol groups all catalysts display a broad adsorption band in the 3700–3400 cm^−1^ region. Usually broad absorption in this region is due to the stretching vibrations of hydrogen-bonded hydroxyl groups.[28] This underlying broad band in this region is more prominently present in Al-Zr-TUD-1 catalysts than it is in their monometallic counterparts. For Al-Zr-TUD-1 catalysts a shoulder at 3580 cm^−1^ is clearly distinguishable.

The strong bridged hydroxyl groups (Brønsted acid site) at around 3613 cm^−1[29]^ as seen in zeolites could not be distinguished in any of the mesoporous TUD-1 samples with incorporated aluminium; which is typical for mesoporous materials as they possess lower Brønsted acidity. Furthermore, due to their amorphous nature the presence of Brønsted acid sites is overshadowed by different hydrogen-bonded hydroxyl groups in the 3700–3400 cm^−1^ region. However, the presence of Brønsted acid sites of zeolitic strength should not be excluded. In addition to catalytic experiments performed with amorphous aluminosilicates proving the existence of Brønsted acid sites of zeolitic strength, further spectroscopic evidence has been provided in a recent publication.[30] However, their density is much lower than that which can be found in zeolites.

**Figure 6:**
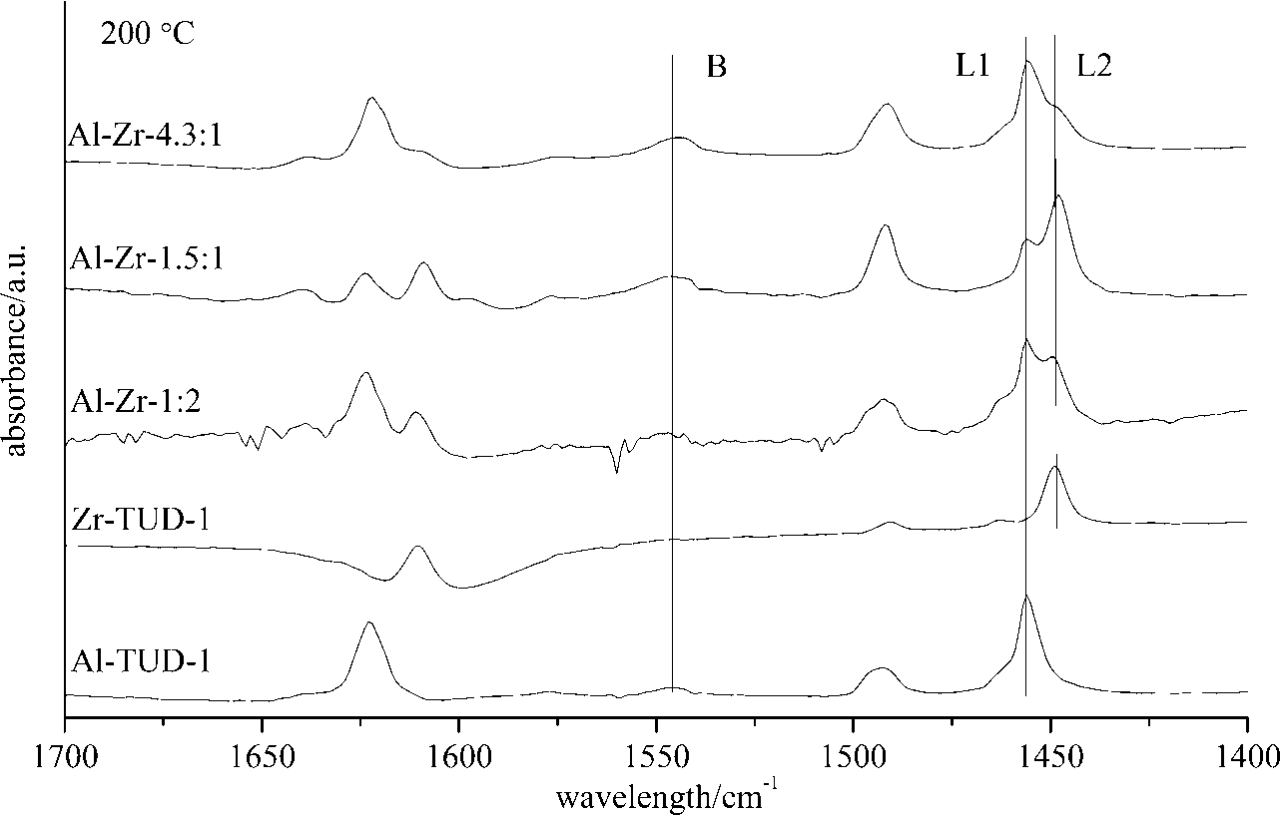
FT-IR spectra after pyridine desorption at 200°C of different TUD-1 catalysts with Si/M ratio of approximately 25: Al-Zr-4.3:1, Al-Zr-1.5:1 Al-Zr-1:2, Zr-TUD-1 and Al-TUD-1. B stands for Brønsted acid site at 1545 cm^−1^ and Lewis acid sites L1 (associated with aluminium) and L2 (associated with zirconium) at respectively 1455 and 1448 cm^−1^.

**Pyridine FT-IR spectroscopic data**: To differentiate between Brønsted and Lewis acid sites in the Al-Zr-TUD-1 catalysts, pyridine FT-IR spectroscopy was employed (Figure 6). Pyridine is more reliable probe molecule than ammonia, since the IR absorption bands do not overlap. Moreover, ammonia decomposes even at rather low temperatures, when adsorbed onto catalysts.[31]

For the Al-TUD-1 sample, the presence of Lewis acid sites is indicated by bands at 1455 (L1) and 1623 cm^−1^. However these bands are not symmetrical and show the presence of other Lewis acid sites at 1460 and 1620 cm^−1^. The presence of these bands has already been reported for H-beta, other zeolites and silica-alumina.[32] The IR band at 1460 cm^−1^ was assigned to iminium ions, formed by attack of protons on the pyridine complex bound to Lewis acid sites. Brønsted acidity is clearly present in Al-TUD-1 as indicated by the band at 1545 cm^−1^ (B).

For Zr-TUD-1 the strength of the Lewis acid site at 1448 cm^−1^ (L2) accompanied by a band at 1612 cm^−1^ seems to be lower than is the case for Al-TUD-1. While for Al-TUD-1 temperatures above 400 °C are needed to desorb pyridine, for Zr-TUD-1 temperatures above 200 °C are already sufficient. Based on this we can conclude that Lewis acidity imparted by partial exchange of aluminium with zirconium generates weaker Lewis acid sites. Brønsted acid sites seem to be absent at outgassing temperatures of 200 °C, which is in line with earlier reports on the synthesis of Zr-TUD-1 leading to framework incorporation of tetravalent zirconium.[17]

Al-Zr-TUD-1 samples show the presence of all the bands present in Al-TUD-1 and Zr-TUD-1. Varying the Al/Zr ratio, but keeping the Si/M ratio constant, leads to variation of the ratio of different Lewis acid sites L1/L2. However, the relation between Lewis acid sites L1 and L2 in Al-Zr-TUD-1 samples does not follow the ratio between the different metals incorporated into the TUD-1 matrix.

The Lewis/Brønsted ratio in all three samples varies (Table 1). For the bimetallic samples containing large amounts of aluminium the Lewis/Brønsted ratio is smaller than that found in Al-TUD-1. However, the sample with the highest amount of zirconium incorporated, Al-Zr-1:2, has a larger Lewis/Brønsted ratio than Al-TUD-1, 3.21 compared to 2.41. Overall, five catalysts with varying Lewis/Brønsted ratios are thus available.

**CO FT-IR spectroscopic data**: CO is a weak base that adsorbs end-on through the carbon on polarised sites.[29] In contrast to more basic ammonia and pyridine, CO is capable of differentiating between sites of very similar acid strength. Its small size, weak basicity, low reactivity (at low temperatures) and sensitivity make it ideal for investigation of samples with both Brønsted and Lewis acid sites.[33]

**Figure 7:**
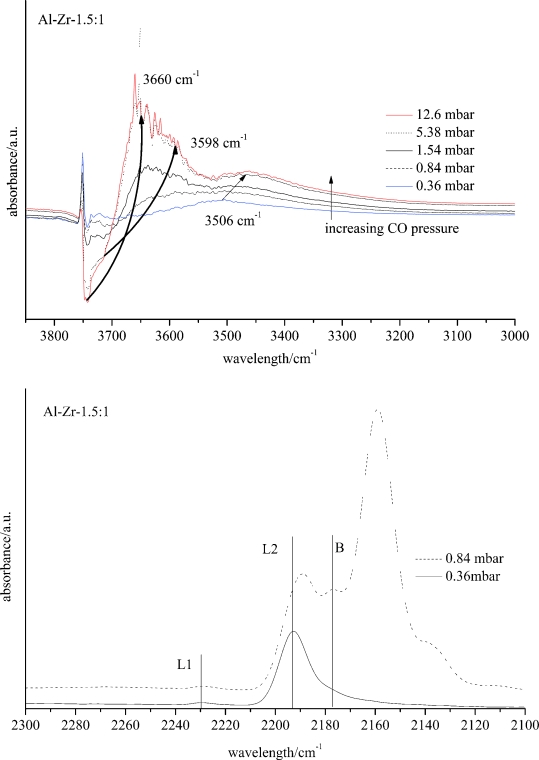
FT-IR difference spectra following CO adsorption obtained at 77 K of Al-Zr-1.5:1 sample: Top: *ν*_(OH)_ region [the spectra are presented as difference plots: from the measured spectra after adsorption of CO, a spectrum of the corresponding pre-treated catalyst has been subtracted—a positive contribution represents peaks than are growing as a result of CO adsorption whereas negative contributions represent peaks that are reduced in intensity upon CO adsorption]. Bottom: the *ν*_(CO)_ region in which Brønsted acid sites are marked by capital letter B, while Lewis acid sites corresponding to different metals are marked by capital letters L1 for aluminium and L2 for zirconium.

Due to the similarities of the shifts of *ν*_OH_ and *ν*_CO_ modes of all Al-Zr-TUD-1 materials and monometallic TUD-1 catalysts only the Al-Zr-1.5:1 FT-IR spectra are discussed here (Figure 7, *ν*_OH_ and *ν*_CO_ modes of other catalysts, see Supporting information Figure S5 and S6).

In the difference spectra of *ν*_OH_ mode of Al-Zr-1.5:1 at very low pressures of CO a broad band appears at 3506 cm^−1^, without development of a clear negative band. At such low pressures, CO will first interact with stronger acid sites as Brønsted acid sites and hydroxyl groups stronger than isolated silanol groups. The broadness of this band suggests heterogeneity of the sample. Even though no clear negative band has developed the red shift of this band (Δ*ν*_OH_) relative to isolated silanol groups is equal to 241 cm^−1^. This value lies between the Δ*ν*_OH_ for silanol groups (90 cm^−1^) and Δ*ν*_OH_ values for strong Brønsted acid sites found in zeolites (300 cm^−1^), implying the presence of medium strong Brønsted acid sites.[34, 35] Isolated silanol groups at these low pressures are unperturbed and appear as a sharp peak at 3750 cm^−1^. Isolated Si–OH groups, usually encountered at 3746 cm^−1^, display a blueshift upon cooling to 3750 cm^−1^, a known temperature effect.[29]

With gradual increase of CO pressure the sharp band at 3750 cm^−1^ associated with weak silanol groups gradually decreases and is accompanied by development of a positive band at 3660 cm^−1^ with a shoulder at 3598 cm^−1^, once all isolated silanol groups are saturated. At the same time negative bands at 3746 and 3741 cm^−1^ develope, with a shoulder at 3716 cm^−1^. The redshift of isolated silanol groups Δ*ν*_OH_ is equal to 87 cm^−1^, which is common for silanol groups.[33, 34] The broad band at 3506 cm^−1^ associated with Brønsted acid sites and strong hydroxyl groups undergo a redshift to 3468 cm^−1^, owing to the solvent effect.[36] The very sharp peak developed at 3650 cm^−1^ at high CO pressure is related to the measuring equipment.

The accompanied *ν*_CO_ mode at low pressure of CO shows two bands appearing at 2229 and 2193 cm^−1^, the latter band having much higher intensity. The blueshift of CO (Δ*ν*_CO_) relative to the free molecule (*ν*_CO_ liquid is 2138 cm^−1^) is 91 cm^−1^ for the band at 2229 cm^−1^, which is usually associated with strong Lewis acid sites related to highly coordinatively unsaturated extra framework Al^3+^.[37, 38] The low intensity of this band indicates that the amount of extra framework Al species is quite low. The band at 2193 cm^−1^, which has a much higher intensity, is associated with CO adsorbed on Zr^4+^ Lewis acid sites, based on CO FT-IR measurements on Zr-TUD-1 and literature values.[39, 40] Lewis acid sites related to Zr^4+^ with a lower frequency shift (Δ*ν*_CO_=58 cm^−1^) are weaker than Lewis acid sites associated with extra framework Al^3+^. This reconfirms the results of the pyridine FT-IR.

With the increase of CO pressure additional bands become evident. Between the high intensity band at 2159 cm^−1^ (generally associated to CO adsorbing to silanol groups) and 2191 cm^−1^ band (shifted due to solvent effect) a new band appears at 2177 cm^−1^ that can be assigned to Brønsted acid sites according to CO FT-IR measurements performed on Al-TUD-1 and literature values.^[41, 42]^ With further increase of CO pressure, bands associated with liquid like CO at 2138 cm^−1^ develop. A very weak band at 2110 cm^−1^ is present as well and has been explained by CO interacting with pairs of ions through oxygen ends.[37]

Substantial shifts in the position of Brønsted acid sites in different Al-Zr-TUD-1 catalysts have not been observed as would be expected if synergistic interaction between Lewis and Brønsted acid sites were present (Table 3).

**Table 3:** Summary of the FT-IR data on the TUD-1 materials: *ν*_OH_ and *ν*_CO_ modes upon CO adsorption at 77 K for Zr-TUD-1, Al-Zr-4.3:1, Al-Zr-1.5:1, Al-Zr-1:2 and Al-TUD-1

	*ν*_OH_ [cm^−1^][a]	Δ*ν*_OH_ [cm^−1^][a]	*ν*_CO_ [cm^−1^] Al^3+^	*ν*_CO_ [cm^−1^] Zr^4+^	*ν*_CO_ [cm^−1^] Si-OH-Al
Zr-TUD-1	3514	233	–	2200	–
Al-Zr-4.3:1	3524	223	2227	2190	2177
Al-Zr-1.5:1	3506	241	2229	2193	2177
Al-Zr-1:2	3525	222	2232	2193	–
Al-TUD-1	3506	241	2230	–	2177

[a] *ν*_OH_ mode of a broad band appearing at low CO pressure in the difference spectra.

Overall extensive spectroscopic investigation of the surface of different Al-Zr-TUD-1 catalysts based on pyridine FT-IR led to the conclusion that partial exchange of aluminium by zirconium in Al-TUD-1 leads to different proportions of Lewis and Brønsted acid sites, but not necessarily to the increase of their strength according to CO FT-IR.

**Performance of Al-Zr-TUD-1 as a catalyst**: The best way to probe whether synergy between Lewis and Brønsted acid sites in Al-Zr-TUD-1 catalysts exists is to employ them in different Lewis and/or Brønsted acid catalysed reactions. In that way the presence of different kinds of acid sites or increase in their strength or amount due to mutual incorporation of aluminium and zirconium in the TUD-1 matrix should reflect itself in increased catalytic activity: synergy. For that reason the catalysts were tested in the Lewis acid catalysed Meerwein–Ponndorf–Verley (MPV) reduction and the C–C bond formation reactions with the Prins reaction. This reaction is catalysed both by Lewis and Brønsted acids and it was performed intermolecularly (nopol synthesis) and intramolecularly (isopulegol synthesis).

**Scheme 1:**
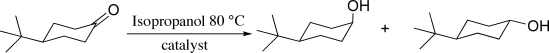
Meerwein–Ponndorf–Verley reduction of 4-*tert*-butylcyclohexanone with isopropanol.

**Scheme 2:**
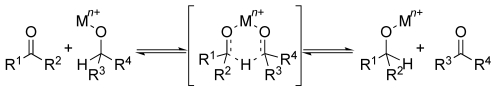
Mechanism of the Meerwein–Ponndorf–Verley reduction.

**Figure 8:**
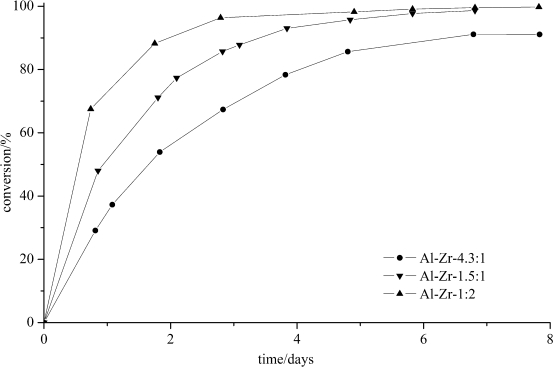
Reduction of 4-*tert*-butylcyclohexanone (2 mmol) in isopropanol (4 mL) at 80°C in the presence of 1,3,5-triisopropylbenzene (0.1 mL) as an internal standard, using different bimetallic catalysts (50 mg) with Si/M ratios of approximately 25: Al-Zr-4.3:1, Al-Zr-1.5:1 and Al-Zr-1:2.

**Figure 9:**
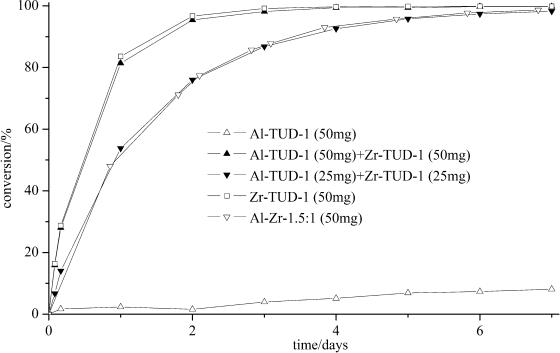
Comparison of bimetallic Al-Zr-1.5:1 catalyst with Al-TUD-1 and Zr-TUD-1 and their physical mixture in reduction of 4-*tert*-butylcyclohexanone (2 mmol) in isopropanol (4 mL) at 80°C in the presence of 1,3,5-triisopropylbenzene (0.1 mL) as an internal standard, using different amounts of activated catalysts. In all reactions the *trans*:*cis* ratio of 4-*tert*-butylcyclohexanol was always around 87:13.

From the study of different Al-Zr-TUD-1 catalysts in the MPV reduction of 4-*tert*-butylcyclohexanone (Scheme 1), it is clear that the increase in amount of zirconium present in the catalysts leads to an increase of conversion (Figure 8). Using aluminium-rich Al-Zr-4.3:1, 30 % conversion was obtained after 24 h compared to 64 % obtained with the Zr-rich catalyst, Al-Zr-1:2. The more zirconium present the more active the catalyst in this Lewis acid catalysed reaction. Comparison of Al-Zr-1.5:1 with monometallic Al-TUD-1 and Zr-TUD-1 and their physical mixtures (Figure 9) demonstrates absence of synergy between Lewis and Brønsted acid sites in the Lewis acid catalysed MPV reduction of 4-*tert*-butylcyclohexanone; the physical mixture showed the same catalytic behaviour as the bimetallic catalyst. The MPV reduction is catalysed by coordination of an alcohol and ketone or an aldehyde to the Lewis acid forming a six-membered transition state enabling a carbon-to-carbon hydride transfer (Scheme 2).[17, 43–45] The more Lewis acid sites a catalysts possesses the more active is the catalyst.

**Scheme 3:**
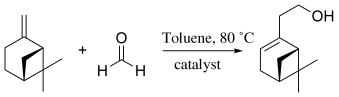
Intermolecular Prins nopol synthesis from β-pinene and paraformaldehyde in toluene at temperature of 80°C.

**Figure 10:**
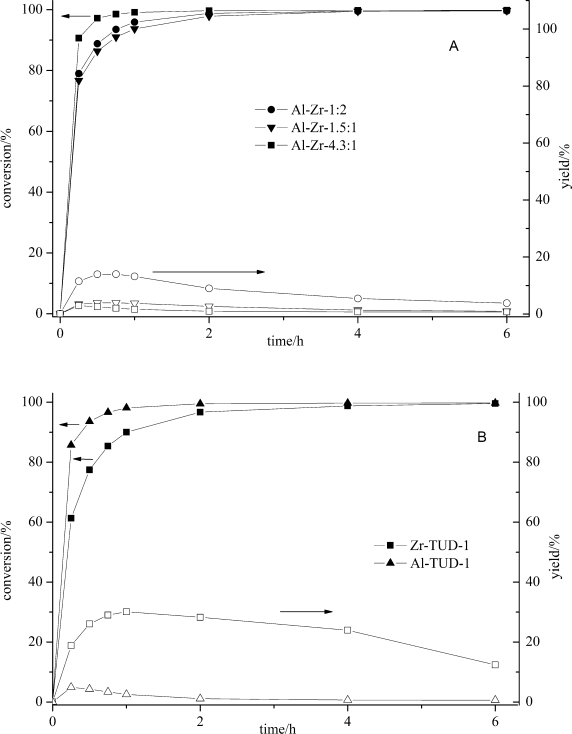
Nopol synthesis performed in toluene (4 mL) at 80°C using paraformaldehyde (2 mmol), 1,3,5-triisopropylbenzene (0.5 mmol) as internal standard, β-pinene (1 mmol) and activated catalyst (50 mg). A) Results obtained with bimetallic catalysts Al-Zr-1:2, Al-Zr-1.5:1 and Al-Zr-4.3:1. B) Results obtained with monometallic catalysts Zr-TUD-1 and Al-TUD-1.

In the nopol synthesis (Scheme 3), a Brønsted and Lewis acid catalysed Prins reaction, no synergy was observed, either. While all of the catalysts were active in the conversion of β-pinene, the yield of nopol was low (Figure 10). Al-TUD-1 and Al-Zr-4.3:1-TUD-1 had the highest activity (full conversion of β-pinene within 1 h). The highest selectivity towards nopol was around 30 % obtained after 1 h reaction time with Zr-TUD-1 (Figure 10). An increase of reaction time led to product degradation. The major side products in nopol synthesis were, according to GC-MS analysis, isomerisation products such as limonene, camphene and terpinolene.

**Scheme 4:**
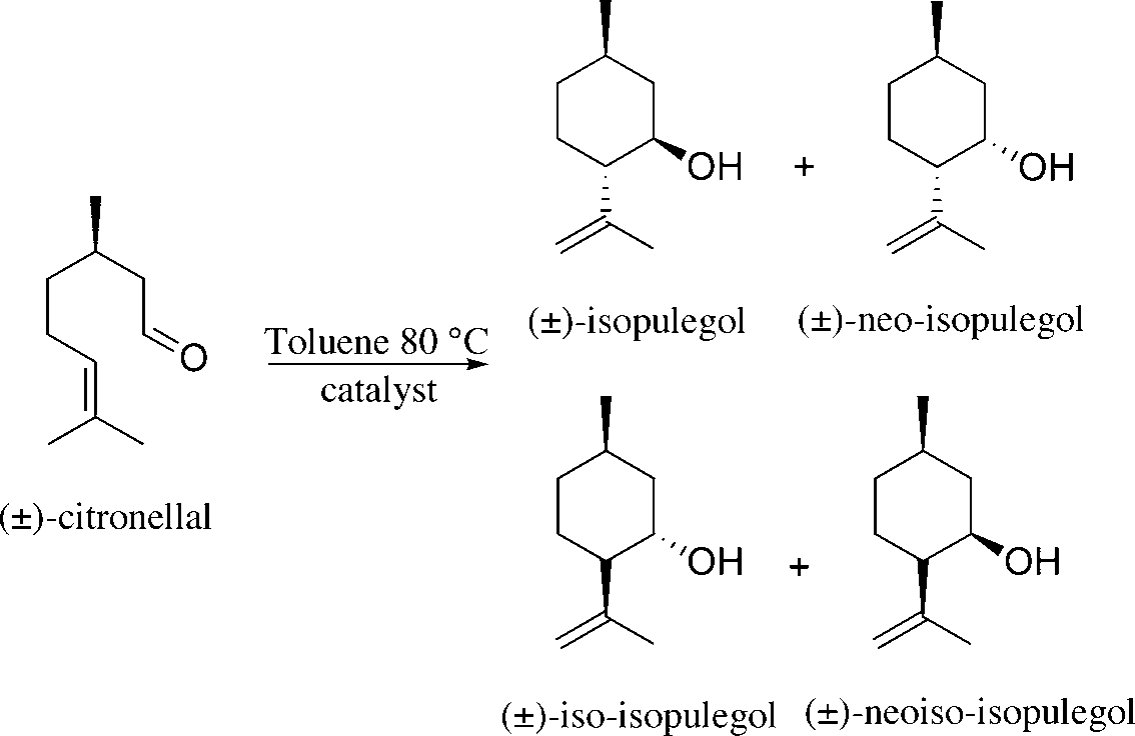
Intramolecular Prins cyclisation of citronellal in toluene at 80°C.

**Figure 11:**
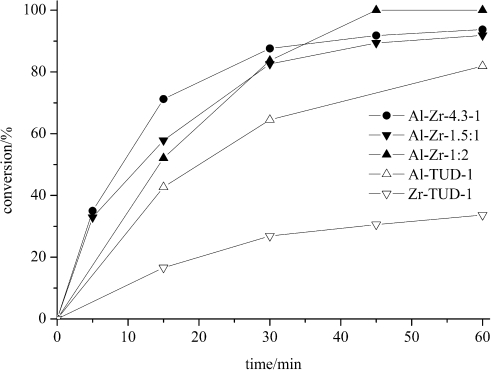
Intramolecular Prins cyclisation of (±)-citronellal (4 mmol) in toluene (5 g) at 80°C using different TUD-1 catalysts (50 mg) with Si/M ratio of approximately 25: Al-Zr-4.3:1, Al-Zr-1.5:1 Al-Zr-1:2, Al-TUD-1 and Zr-TUD-1.

However, excellent results were obtained in Prins cyclisation of (±)-citronellal (Scheme 4). All three samples of Al-Zr-TUD-1 catalysts outperformed their monometallic counterparts Al-TUD-1 and Zr-TUD-1 (Figure 11). This, despite of the fact that both Al-TUD-1 and Zr-TUD-1 possess larger amounts of acid sites according to NH_3_ TPD. Aluminium-rich Al-Zr-TUD-1 catalysts led to high initial rates, but full conversions are not reached as also in the case of the catalyst containing the smallest amount of aluminium, Al-Zr-1:2. Selectivity in all cases was above 95 % and major isomers were (±)-isopulegol and (±)-neo-isopulegol.

**Scheme 5:**
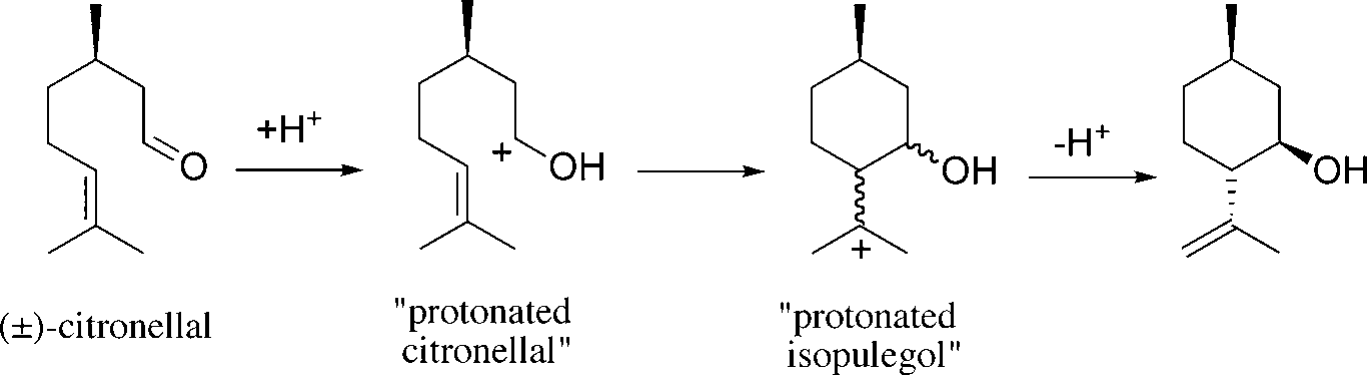
The Brønsted acid catalysed Prins cyclisation of citronellal.

**Scheme 6:**
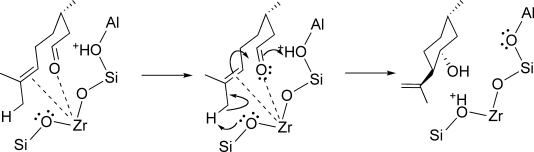
The Brønsted/Lewis acid catalysed Prins cyclisation of citronellal, a new carbon–carbon bond is formed between an alkene and an aldehyde.

Both types of Prins reaction, intramolecular citronellal cyclisation and intermolecular Nopol synthesis, can be catalysed by Brønsted and/or Lewis acid sites. Exclusively Brønsted acid catalysts catalyse the Prins cyclisation via the formation of carbenium ion intermediates (Scheme 5).[46, 47] In the Prins cyclisation of citronellal by means of an intramolecular reaction, a more stable tertiary carbocation is formed. Clearly intramolecular reaction will more readily occur than intermolecular reaction as is the case in Nopol synthesis were the availability of the reacting alkene group at the rather hydrophilic surface (based on the FTIR results in Figure 5) is lower. This difference is even more enhanced for catalysts containing both the Brønsted and Lewis acid sites. Due to activation of both reacting groups, the alkene and the carbonyl group, by Lewis acid sites and the presence of Brønsted acid sites in close proximity, the rate of the reaction is further enhanced as is the case with bimetallic Al-Zr-TUD-1 materials in the Prins cyclisation of citronellal. It was already proposed earlier that the desired heterogeneous catalysts for the cyclisation of citronellal should have strong Lewis and weak Bronsted acid sites.[48] It is believed that citronellal coordinates in an orientation favourable for ring closure were both the oxygen of the aldehyde group, as the electron-rich double bonds are attached to Lewis acid sites, in this case zirconium. In the transition state, protonation of the oxygen atom occurs through neighbouring Brønsted hydroxyl groups from the surface of the support.[48–50] Subsequently, hydrogen is abstracted from the isopropyl group and the ring is closed to form isopulegol (Scheme 6).

Based on these results we can conclude that synergy in Al-Zr-TUD-1 obtained in the Prins cyclisation of citronellal is much more complex and more subtle than can be explained by increase of Brønsted acidity due to incorporation of a different Lewis acid. All three samples of Al-Zr-TUD-1 show synergistic properties in the Prins cyclisation of citronellal, even though the proportions of Lewis acid sites to Brønsted acid sites are different according to pyridine FT-IR. The sample Al-Zr-1:2, with predominately Lewis acidic character has a lower initial rate than more aluminium-rich Al-Zr-TUD-1 catalysts, but eventually reaches full conversion first. Demonstration of synergy could not be repeated for other C–C bond formation reactions. A possible explanation could be that in many of the heterogeneously catalysed reactions, the match between the strength of acid sites and substrates plays an important role. In another study, even though not explicitly mentioned as synergy, a similar observation was made for the same intramolecular Prins reaction. By varying the F/OH ratio during synthesis of nanoscopic magnesium fluorides, unexpected catalytic properties were obtained in the Prins cyclisation of citronellal.[51, 52] In that study it was also spectroscopically proven that the different combinations and variable strength of Lewis and Brønsted acid sites are responsible for increased activity.

Another very important difference between Prins cyclisation and nopol synthesis is the difference between intramolecular and intermolecular reaction. Intramolecular reactions generally proceed much more rapidly and under much milder reaction conditions than their intermolecular counterparts, since the two reacting groups are already in close proximity to one another. Indeed, this is one of the underlying reasons for the great catalytic power of enzymes.[53, 54]

## Conclusion

Al-Zr-TUD-1, a three-dimensional mesoporous material containing varying amounts of aluminium and zirconium, was synthesised by using triethanolamine as complexing agent. Framework incorporation of Al and Zr is evidenced by absence of Al_2_O_3_ or ZrO_2_ phases, as proven by X-ray diffraction, HR-TEM and XPS studies. Extensive FT-IR analysis with pyridine and CO as probe molecules did not allow us to identify synergistic properties due to incorporation of zirconium as a Lewis acid in addition to aluminium generating both Lewis as well as Brønsted acidity.

The search for synergistic properties was therefore performed with catalytic experiments. Synergy was not encountered if Al-Zr-TUD-1 catalysts were applied in Lewis acid catalysed Meerwein–Ponndorf–Verley reduction of 4-*tert*-butylcyclohexanone or the intermolecular Prins nopol synthesis. However, synergistic properties of Al-Zr-TUD-1 catalysts were clearly evidenced in the Prins cyclisation of citronellal, owing to proximity effects of reacting groups in this intramolecular reaction. Along with the excellent properties of TUD-1 type materials allowing framework incorporation of different metals as well as a systematic variation of two metals rather than merely increasing the weight percentage of one metal, while keeping the weight percentage of the second constant. While we do not have yet the right spectroscopic technique in combination with probe molecules to discriminate between the sites so to identify synergistic effects, catalytic reactions can pinpoint synergistic properties. However, it is only possible for specific reactions, namely the Prins cyclisation of citronellal. In another type of Prins reaction (nopol synthesis), a synergistic effect could not be observed, owing to the intermolecular nature of the reaction and hydrophilic surface of the catalyst. In other words, synergistic effects seem to be reaction mechanism dependent.

## Experimental Section

**Catalyst synthesis**: Monometallic Al-TUD-1 and Zr-TUD-1 were synthesised according to previous reports and were described earlier in full detail.[17, 18]

**Al-Zr-TUD-1**: Al-Zr-TUD-1 materials were synthesised by using triethanolamine (TEA, ≥99.0 %, Fluka) as a complexing agent in a one pot surfactant-free procedure based on the sol–gel technique. Al-Zr-TUD-1 materials with constant Si/(Al+Zr) molar ratio of 25 with varying Al/Zr ratios were synthesised by adjusting the molar ratio of SiO_2_/*x* Al_2_O_3_/*y* ZrO_2_/tetraethylammonium hydroxide (TEAOH)/(0.5–1) TEA/(10–20) H_2_O. In a typical synthesis (Al/Zr=1:3), aluminum(III) isopropoxide (0.51 g, 98+ %, Aldrich) and zirconium(IV) propoxide solution (0.32 g, 70 wt % in 1-propanol, Aldrich), dissolved in a 1:1 mixture of isopropanol (HPLC grade, Fisher Chemicals, 0.013 % H_2_O) and absolute ethanol (J. T. Baker, 0.2 % H_2_O), was added to tetraethyl orthosilicate (17.3 g; 98 %, Aldrich). After stirring for a few minutes, a mixture of TEA (12.5 g; ≥99.0 %, Fluka) and water (9.4 g) was added, followed by addition of TEAOH (10.2 g; 35 wt % in H_2_O, Aldrich) under vigorous stirring. The clear gel obtained after these steps was then aged at room temperature for 12–24 h and dried at 98 °C for 12–24 h, followed by hydrothermal treatment in a Teflon-lined autoclave at 180 °C for 4–24 h and final calcination in the presence of air up to 600 °C with a temperature ramp of 1 °C min^−1^. Al-Zr-TUD-1 samples with constant Si*/*(Al+Zr) ratio of approximately 25 and varying Al/Zr ratio of 3, 1 and 0.33 were prepared and are denoted as Al-Zr-4.3:1, Al-Zr-1.5:1 and Al-Zr-1:2, respectively, based on ICP results.

**Catalysts characterisation**: Chemical analysis of Si, Al and Zr were performed in duplet by dissolving the samples in 1 % HF (48 % in H_2_O, 99.99 + % based on metal basis, Aldrich) and 1.25 % H_2_SO_4_ (99.999 %, Aldrich) solution and measuring them with inductively coupled plasma—optical emission spectroscopy (ICP-OES) on a Perkin–Elmer Optima 3000DV instrument. The textural properties of the materials were characterised by volumetric N_2_ physisorption at 77 K using Micromeritics ASAP 2010 equipment. Prior to the physisorption experiment, the samples were dried overnight at 573 K (*p*≤10–2 Pa). From the nitrogen sorption isotherms, the specific surface area S_BET_, the pore diameter *d*_P,BJH_ and the pore volume *V*_P,BJH_ were calculated.

High-resolution transmission electron microscopy (HR-TEM) was performed on a Philips CM30UT electron microscope with a LaB6 filament as the source of electrons operated at 300 kV. Samples were mounted on Quantifoil® carbon polymer supported on a copper grid by placing a few droplets of a suspension of ground sample in ethanol on the grid, followed by drying at ambient conditions.

Powder X-ray diffraction (XRD) patterns were obtained on a Philips PW 1840 diffractometer equipped with a graphite monochromator using Cu_Kα_ radiation.

^27^Al MAS NMR experiments were performed at 9.4 T on a Varian VXR-400 S spectrometer operating at 104.2 MHz with a pulse width of 1 ms. 4 mm Zirconia rotors with a spinning speed set to 6 kHz were used. The chemical shifts are reported with respect to Al(NO_3_)_3_ as external standard at *δ*=0 ppm.

The XPS measurements were performed with a PHI 5400 ESCA provided with a dual Mg/Al anode X-ray source, a hemispherical capacitor analyser and a 5 keV ion-gun. Powdered catalyst samples were pressed into clean indium foil (Alfa Products, purity 99,9975 %) with a thickness of 0.5 mm and subsequently placed on a flat specimen holder. The input lens optical axis to the analyser was at a take off angle of 15° with respect to the sample surface normal. The input lens aperture used was 3.5×1.0 mm. All spectra were recorded with non-monochromated magnesium radiation. The X-ray source was operated at an acceleration voltage of 13 keV and a power of 200 W. A survey spectrum was recorded between 0 and 1000 eV binding energy using pass energy of 71.95 eV and step size of 0.25 eV. The spectra of the separate photoelectron and Si-Auger electron lines were recorded with pass energy of 35.75 eV and step size of 0.2 eV. The Zr-Auger electron line was recorded with pass energy of 89.45 eV and step size of 0.5 eV. The conditions for the spectra are summarised in Table S2 of Supporting Information. The spectra were evaluated with Multipak 8.0 software (Physical electronics). Firstly, the satellite photoelectron lines were substracted from the spectrum. Next the energy scale was aligned adopting a value of 103.5±0.2 eV for the binding energy of the Si 2p photoelectron line present in the Si-TUD-1 carrier implying a binding energy of 532.9±0.2 eV for the O 1s line.[21] Then, the background intensity was subtracted from the spectra using a Shirley method.[55] Afterwards, the spectra were fitted with (symmetrical) mixed Gauss–Lorentz functions by using the linear least-square method to resolve the chemical states of the constituting components. The peaks describing sample Si-TUD-1 were kept fixed during the deconvolution of the Al and Zr loaded catalysts.

Temperature-programmed desorption (TPD) of ammonia was carried out on a Micromeritics TPR/TPD 2900 equipped with a thermal conductivity detector (TCD). The sample (30 mg) was pre-treated at 823 K to remove volatile components. Prior to the TPD measurements the samples were saturated with ammonia gas at 393 K. This procedure was repeated three times. The measurements were only started when as much as possible physisorbed NH_3_ was removed. Desorption of NH_3_ was monitored in the range between 393 and 823 K at a ramp rate of 10 K min^−1^.

Skeletal FTIR spectra were measured in the 1500–600 cm^−1^ region. FTIR spectra of KBr diluted wafers of samples were recorded using a Perkin–Elmer Spectrum One instrument. In total 19 scans were taken with a resolution of 4 cm^−1^.

FT-IR spectra of the OH region were measured in 3900–3000 cm^−1^ region. FTIR spectra of self-supported wafers were recorded with a Thermo Nicolet FT-IR Nexus instrument. Self-supported wafers were pre-treated at 500 °C in three-window cells (CaF_2_) under a flow of He. In total 128 scans were taken with resolution of 4 cm^−1^.

A Perkin–Elmer 2000 FT-IR instrument was used to record FT-IR spectra after pyridine desorption at various temperatures. Self supported catalyst wafers (18–25 mg/16 mm) were pressed at 3 bar pressure applied for 10 s. The wafer was placed inside a glass cell with KBr windows and subsequently evacuated to 10^−6^ bar followed by drying at 300 °C (3 °C min^−1^) for 1 h. The cell was cooled down to room temperature and the IR spectrum was collected. Then the temperature of the cell was raised to 50 °C and the sample was brought into contact with pyridine vapours (3.1 mbar) for 10 min. Afterwards by applying vacuum for 30 min physisorbed and loosely bound pyridine was removed. FT-IR spectra were recorded under vacuum under various conditions by increasing the temperature (3 °C min^−1^) from 50 to 450 °C. For each spectrum 25 scans were recorded with resolution of 4 cm^−1^.

CO adsorption experiments were performed with Perkin–Elmer 2000 FTIR instrument. Self supporting wafers were prepared by applying 3 bar pressure for 10 s. The wafers were placed in a stainless steel IR transmission cell (12–17 mg per 13 mm) equipped with CaF_2_ windows. The cell was evacuated at 10^−8^ bar followed by drying at 300 °C (ramp rate 3 °C min^−1^) for 1 h. Subsequently the wafers were cooled down to 77 K with liquid N_2_. A background spectrum was taken prior to CO exposure. CO was introduced as 10 % CO in He (Linde gas) (0.1 mbar to 30 mbar) at 77 K. For each spectrum 25 scans were recorded with a resolution of 4 cm^−1^.

**Catalysts testing**: The catalytic experiments were performed in dried glassware using Schlenk techniques. The anhydrous solvents were used as received.

For the Meerwein-Ponndorf-Verley reductions, 4-*tert*-butylcyclohexanone (2 mmol; 99 %, Aldrich), isopropanol (4 mL; 99.5 %, Aldrich) and 1,3,5-triisopropylbenzene (0.1 mL; 96 %, Aldrich, internal standard) were loaded in the Schlenk flask containing the activated Al-Zr-TUD-1 catalyst (50 mg; 600 °C, 10 h, 1 °C min^−1^). The reaction flask was immersed into a preheated oil bath at 80 °C. Periodically samples were withdrawn (20 μL) and analysed on a GC (Shimadzu GC-17 A gas chromatograph) equipped with a 25 m×0.32 mm×0.25 μm chiral column ChrompackTM Chirasil-Dex CB and a FID detector. The reactants and products were identified by comparison with the retention times of authentic samples and additionally by NMR spectroscopy as described earlier.[17] Employing an isotherm (120 °C) following retention times were recorded: 1,3,5-triisopropylbenzene (3.86 min), 4-*tert*-butylcyclohexanone (4.86 min), *cis*-4-*tert*-butylcyclohexanol (5.4 min) and *trans*-4-*tert*-butylcyclohexanol (5.8 min).

In the intermolecular nopol synthesis catalysts (50 mg) were dried at 120 °C under vacuum for 1 h. To the dried catalyst materials paraformaldehyde (2 mmol, 0.07 g; 95 %, Merck) was added followed by dry toluene (4 mL; 99.8 %, Aldrich) and 1,3,5-triisopropylbenzene (0.5 mmol, 0.10 g; 96 %, Aldrich) as internal standard. Finally, β-pinene (1 mmol, 0.14 g; 99 %, Aldrich) was added. The reaction was started by submerging the reaction mixture into a preheated oil bath at 80 °C. Samples (20 μL) were analysed by GC (Shimadzu GC-2014 gas chromatograph) equipped with a 50 m×0.53 mm×1.0 μm CP-Sil 5CB column and using a FID detector. Employing an isotherm (110 °C) following retention times were recorded: β-pinene (2.01 min), nopol (7.24 min) and 1,3,5-triisopropylbenzene (10.40 min).

The intramolecular Prins reaction was performed as reported earlier.[50] The catalysts were dried at 100 °C overnight. (±)-Citronellal (4 mmol; ≥95.0 %, Aldrich) and solvent (5 g), toluene (99.8 %, Aldrich) or *tert*-butanol (≥99.5 %, Aldrich), were added to the catalyst (50 mg). The reactions were performed at 80 °C or at room temperature. Samples were withdrawn periodically and analysed by GC (Agilent’s HP5 column). The different isomers were identified by ^1^H NMR spectroscopy.
