# Supplementary material for: On the Synergistic Catalytic Properties of Bimetallic Mesoporous Materials Containing Aluminum and Zirconium: The Prins Cyclisation of Citronellal
Source: Chemistry. 2011 Jan 21;17(7):2077–88. doi: 10.1002/chem.201002909 (PMC3072521; doi:10.1002/chem.201002909)
Supplement: Supplementary file 1 [file chem0017-2077-SD1.pdf]

CHEMISTRY
A EUROPEAN JOURNAL

Supporting Information

© Copyright Wiley-VCH Verlag GmbH & Co. KGaA, 69451 Weinheim, 2011

On the Synergistic Catalytic Properties of Bimetallic Mesoporous Materials Containing Aluminum and Zirconium: The Prins Cyclisation of Citronellal

Selvedin Telalović,[a] Anand Ramanathan,[b] Jeck Fei Ng,[c] Rajamanickam Maheswari,[d] Cees Kwakernaak,[e] Fouad Soulimani,[f] Hans C. Brouwer,[e] Gaik Khuan Chuah,[c] Bert M. Weckhuysen,[f] and Ulf Hanefeld*[a]

chem_201002909_sm_miscellaneous_information.pdf

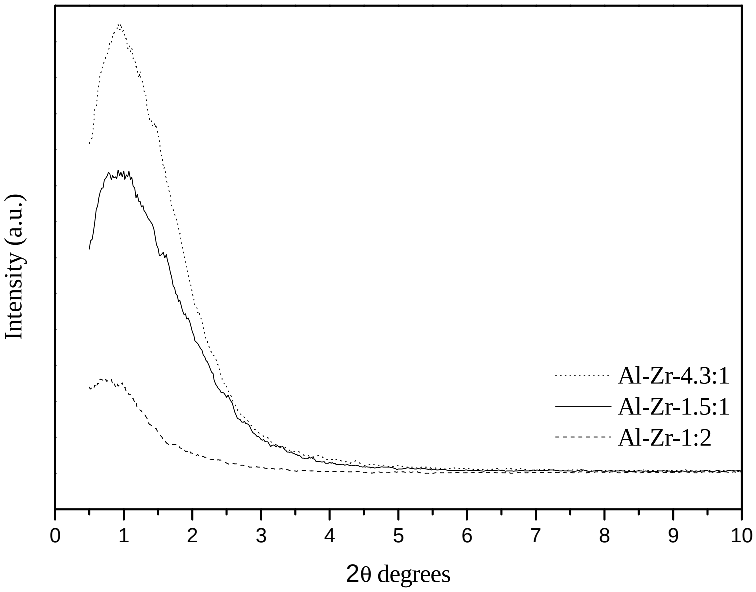

Figure S1. Al-Zr-TUD-1 catalysts with varying ratios of Al : Zr (4.3 : 1, 1.5 : 1 and 1: 2) but constant Si/M ratio of approx. 25 show an intense peak at low angle (0.8-1 2θ) in their X-ray powder diffraction patterns. The higher the percentage zirconium incorporated, the lower the intensity of the peak and therefore the degree of order of the corresponding Al-Zr-TUD-1. No evidence of crystalline ZrO_2 or Al_2O_3 phases was found in the X-ray diffractograms, suggesting that both metals were incorporated into the framework as expected.

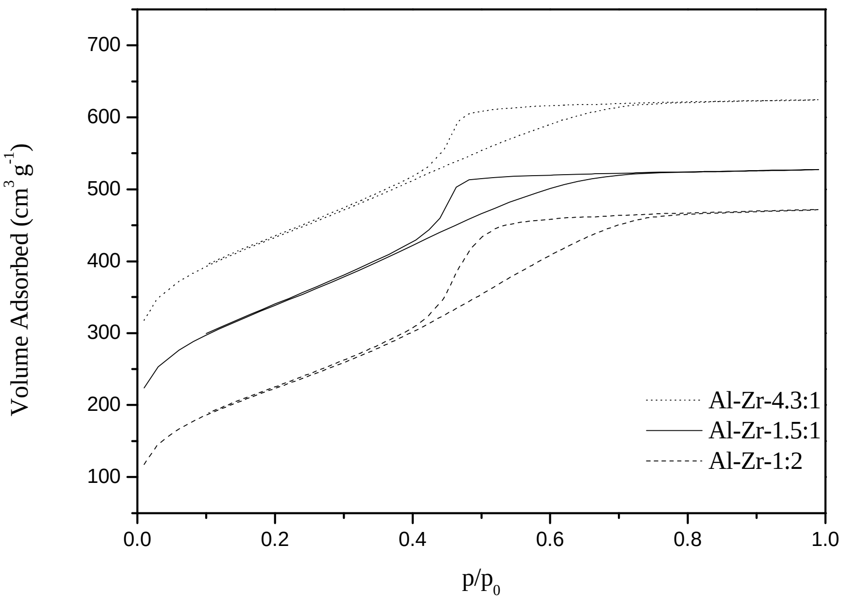

Figure S2. Isotherms of bimetallic TUD-1 catalysts with Si/M ratio of approx. 25 and Al-Zr-4.3:1, Al-Zr-1.5:1 and Al-Zr-1:2 obtained from N_2 physisorption analysis.

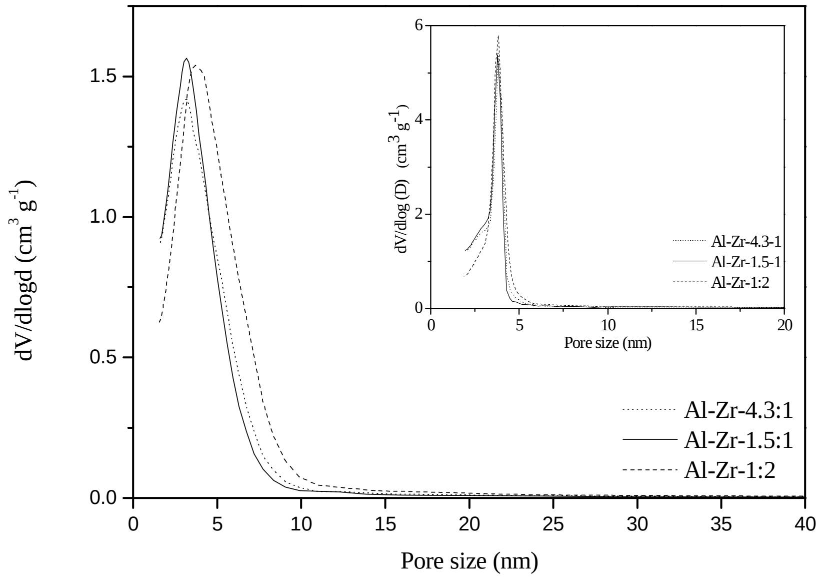

Figure S3. Pore size distribution calculated from adsorption and desorption branch (inset) using the Barret-Joyner-Halenda (BJH) model of bimetallic TUD-1 catalysts with Si/M ratio of approx. 25 and Al-Zr-4.3:1, Al-Zr-1.5:1 and Al-Zr-1:2.

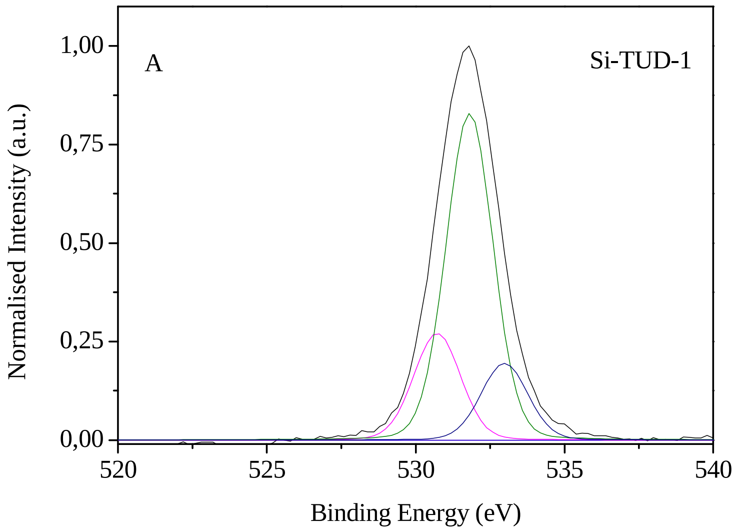
A
Si-TUD-1
Normalised Intensity (a.u.)
1,00
0,75
0,50
0,25
0,00
520
525
530
535
540
Binding Energy (eV)

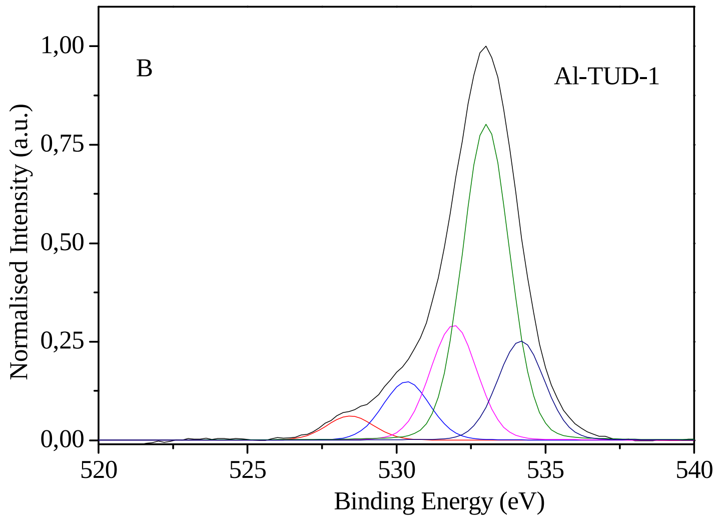
B
Al-TUD-1
Normalised Intensity (a.u.)
1,00
0,75
0,50
0,25
0,00
520
525
530
535
540
Binding Energy (eV)

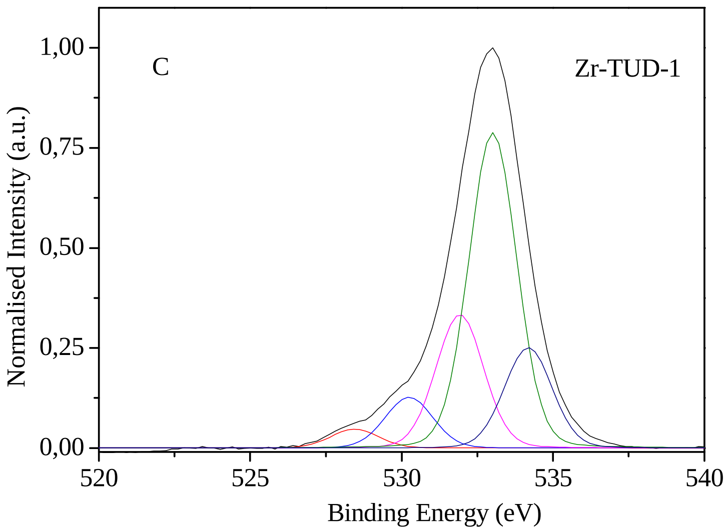
C
Zr-TUD-1
Normalised Intensity (a.u.)
Binding Energy (eV)
1,00
0,75
0,50
0,25
0,00
520
525
530
535
540

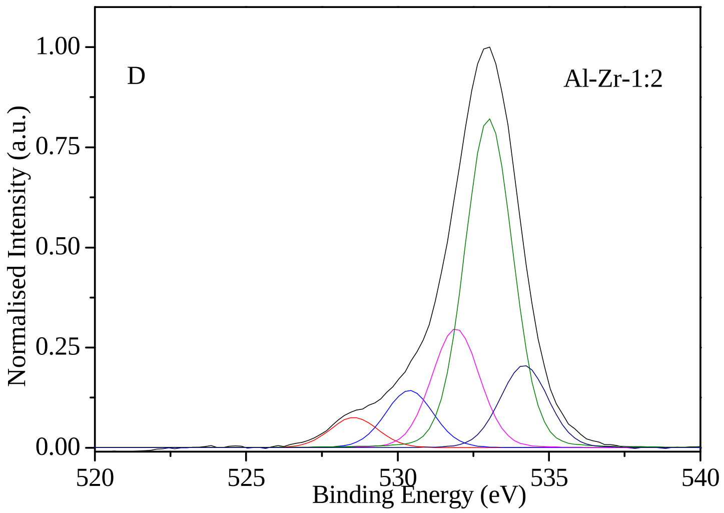
D
Al-Zr-1:2
Normalised Intensity (a.u.)
Binding Energy (eV)
1.00
0.75
0.50
0.25
0.00
520
525
530
535
540

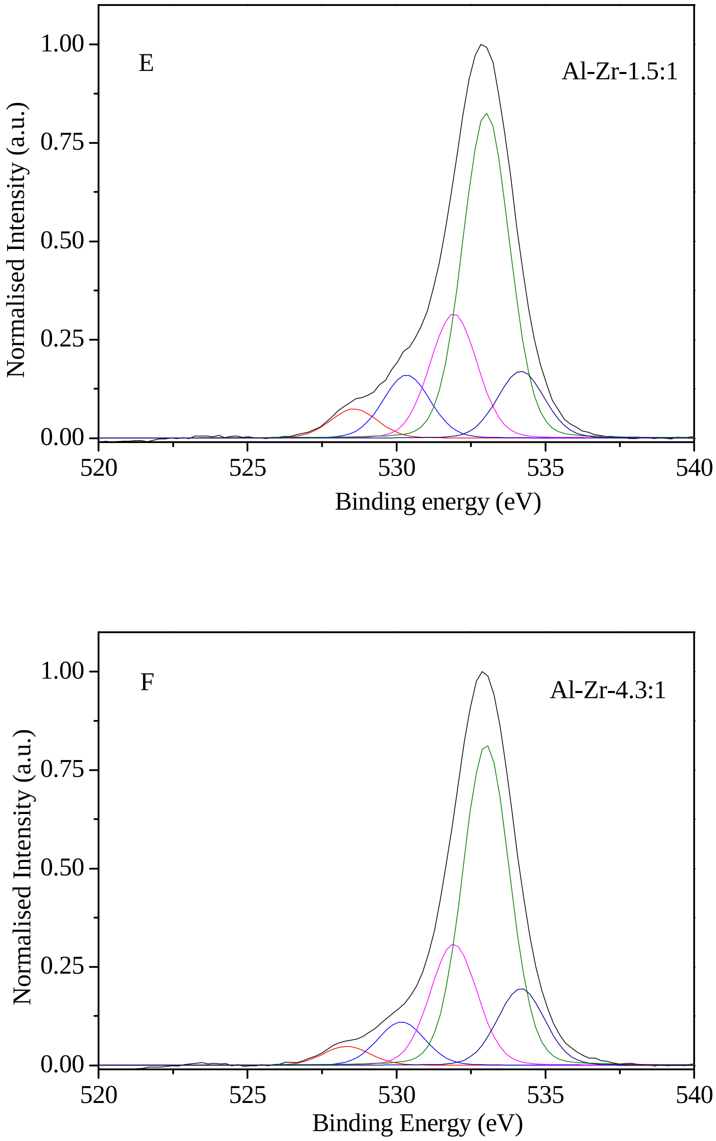

Figure S4. Deconvoluted O *1s* spectra obtained by XPS analysis of (A) Si-TUD-1; (B) Al-TUD-1; (C) Zr-TUD-1; (D) Al-Zr-1:2; (E) Al-Zr-1.5:1; (F) Al-Zr-4.3:1.

Table S1. XPS Analysis: The area fraction of the O *1s* in percent of the total area is given for each catalyst.

Spectral Line	Si-TUD-1	Al-TUD-1	Zr-TUD-1	Al-Zr-4.3:1	Al-Zr-1.5:1	Al-Zr-1:2	Remark
O *1s* (1)	-	4.0	3.1	3.3	4.8	4.9	MO_x
O *1s* (2)	-	9.5	8.2	7.5	10.4	9.3	MO_x
O *1s* (3)	20.9	18.8	21.6	20.8	20.4	19.2	TUD-1
O *1s* (4)	64.0	51.5	50.9	55.2	53.4	53.3	TUD-1
O *1s* (5)	15.1	16.2	16.2	13.2	11.0	13.3	TUD-1, CO, H_2O

Table S2. Analysis region of the photo-electron and Auger lines recorded with XPS using Al-K_α radiation.

Spectral Line designation	Sensitivity factors		Analysis region (eV)
	Area	Height	
C *1s*	0.314	0.314	275 - 300
O *1s*	0.733	0.733	520 - 550
Al *2p*	0.256	0.249	68 - 88
Si *2p*	0.368	0.341	95 - 120
Zr *3d*	2.767	1.977	174 - 199
Al *KLL*			(-330) – (-230)
Si *KLL*			(-370) – (-330)
Zr *LMM*			(-630) – (-530)

Table S3. The atomic fraction in percent of the main constituent elements for each catalyst material and the atomic ratio of Al to Zr and Si to Zr obtained by XPS analysis.

Atomic fraction	Zr-TUD-1	Al-TUD-1	Al-Zr-4.3:1	Al-Zr-1.5:1	Al-Zr-1:2
O	72.6	70.8	71.1	74.5	71.1
Al	-	1.3	1.0	0.8	0.7
Si	26.5	27.9	27.5	24.4	27.7
Zr	0.9	-	0.4	0.3	0.5
Si/Al	-	21	28	31	40
Si/Zr	29	-	69	81	55

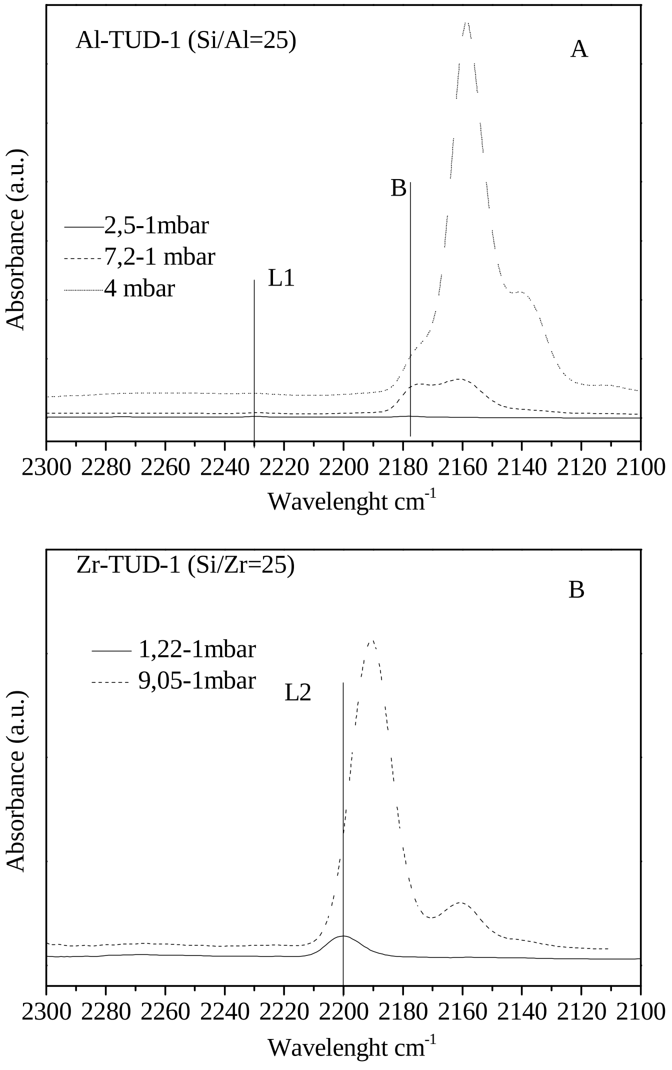

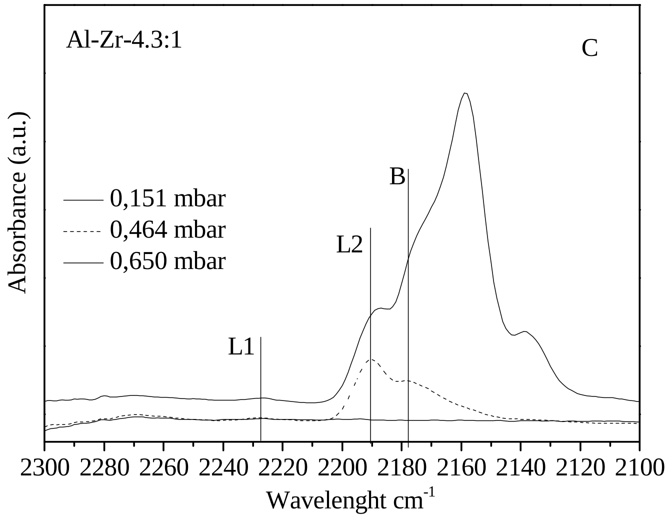

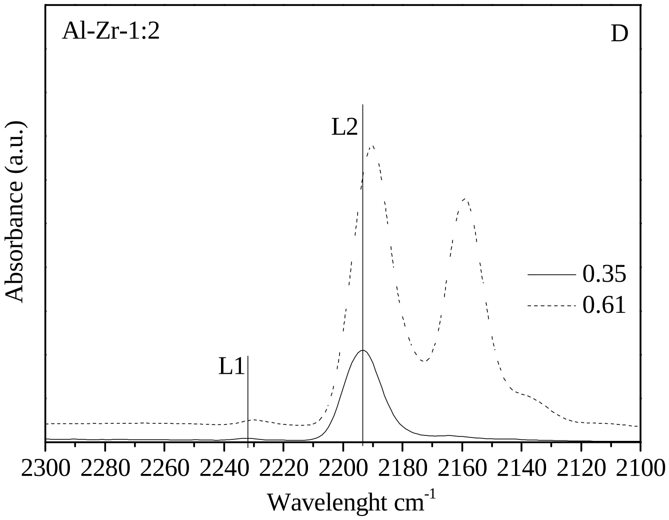

Figure S5. FT-IR Difference spectra following CO adsorption obtained at 77 K of the $\nu_{(CO)}$ region . The $\nu_{(CO)}$ region, were Brønsted acid sites are marked by capital letter B while Lewis acid sites corresponding to different metals are marked by capital letters L1 for aluminium and L2 for zirconium for (A): Al-TUD-1; (B) Zr-TUD-1; (C) Al-Zr-4.3:1 and (D) Al-Zr-1:2.

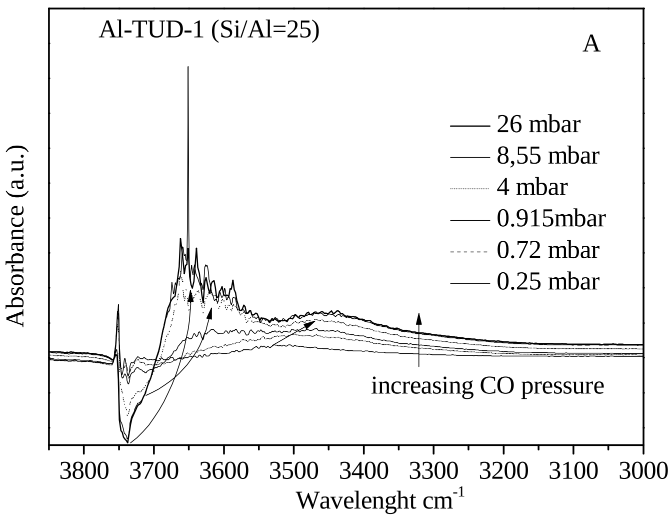
Al-TUD-1 (Si/Al=25)
A
26 mbar
8,55 mbar
4 mbar
0.915mbar
0.72 mbar
0.25 mbar
increasing CO pressure
Absorbance (a.u.)
3800 3700 3600 3500 3400 3300 3200 3100 3000
Wavelenght cm-1

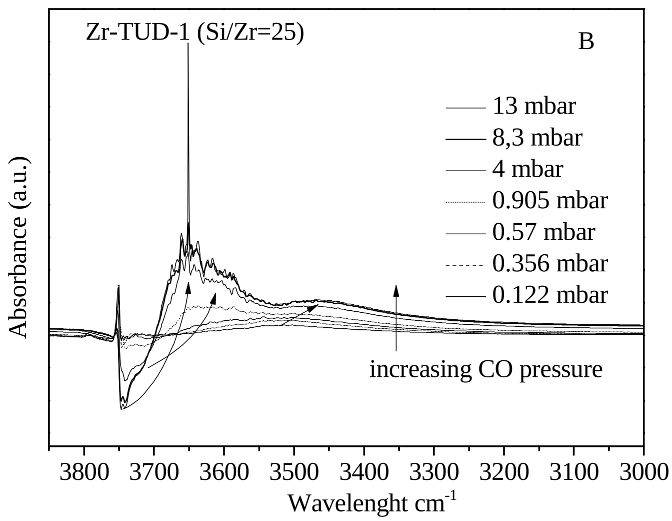
Zr-TUD-1 (Si/Zr=25)
B
13 mbar
8,3 mbar
4 mbar
0.905 mbar
0.57 mbar
0.356 mbar
0.122 mbar
increasing CO pressure
Absorbance (a.u.)
3800 3700 3600 3500 3400 3300 3200 3100 3000
Wavelenght cm-1

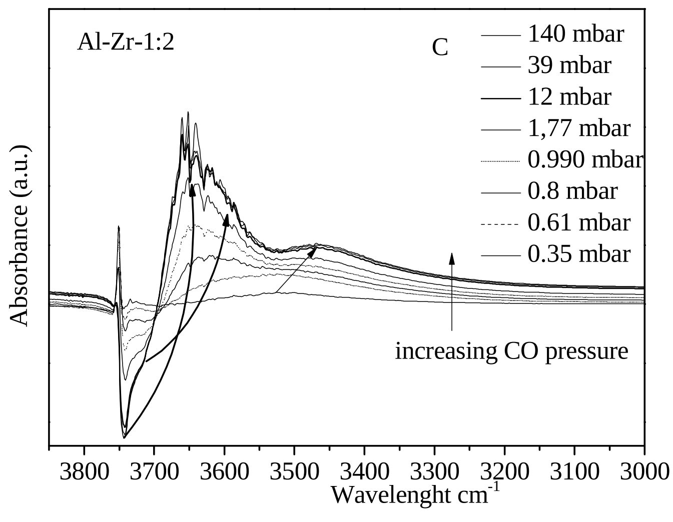
Al-Zr-1:2
C
140 mbar
39 mbar
12 mbar
1,77 mbar
0.990 mbar
0.8 mbar
0.61 mbar
0.35 mbar
increasing CO pressure
Absorbance (a.u.)
3800 3700 3600 3500 3400 3300 3200 3100 3000
Wavelenght cm-1

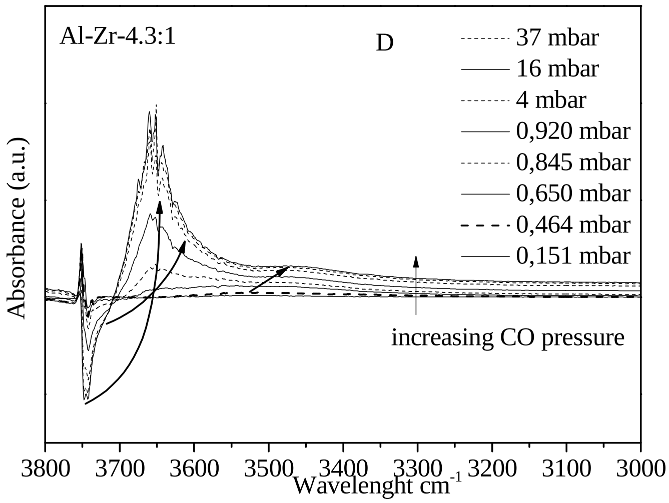

Figure S6. FT-IR Difference spectra following CO adsorption obtained at 77 K of the $\nu_{(OH)}$ region . The spectra are presented as difference plots: from the measured spectra after adsorption of CO, a spectrum of the corresponding pre-treated catalyst has been subtracted. A positive contribution represents peaks than are growing as a result of CO adsorption whereas negative contributions represent peaks that are reduced in intensity upon CO adsorption. (A): Al-TUD-1; (B) Zr-TUD-1; (C) Al-Zr-1:2 and (D) Al-Zr-4.3:1.
